# Inhibition of microsomal prostaglandin E synthase-1 ameliorates acute lung injury in mice

**DOI:** 10.1186/s12967-021-03016-9

**Published:** 2021-08-09

**Authors:** Malarvizhi Gurusamy, Saeed Nasseri, Dileep Reddy Rampa, Huiying Feng, Dongwon Lee, Anton Pekcec, Henri Doods, Dongmei Wu

**Affiliations:** 1 Department of BIN Convergence Technology, Chonbuk National University, Jeonju, South Korea; 2 Boehringer Ingelheim Pharma GmbH & Co. KG, Biberach an der Riss, Germany; 3 Department of Research, Mount Sinai Medical Center, Miami Beach, FL USA; 4 Present Address: Cellular and Molecular Research Center, Birjand University of Medical Sciences, Birjand, Iran

**Keywords:** Leukocyte infiltration, Lung injury, mPGES-1, Vascular permeability, BI 1029539, GS-248, Celecoxib, Sepsis

## Abstract

**Background:**

To examine the effects of BI 1029539 (GS-248), a novel selective human microsomal prostaglandin E synthase-1 **(**mPGES-1) inhibitor, in experimental models of acute lung injury (ALI) and sepsis in transgenic mice constitutively expressing the mPGES1 (Ptges) humanized allele.

**Methods:**

Series 1: Lipopolysaccharide (LPS)-induced ALI. Mice were randomized to receive vehicle, BI 1029539, or celecoxib. Series 2: Cecal ligation and puncture-induced sepsis. Mice were randomized to receive vehicle or BI 1029539.

**Results:**

Series 1: BI 1029539 or celecoxib reduced LPS-induced lung injury, with reduction in neutrophil influx, protein content, TNF-ɑ, IL-1β and PGE_2_ levels in bronchoalveolar lavage (BAL), myeloperoxidase activity, expression of mPGES-1, cyclooxygenase (COX)-2 and intracellular adhesion molecule in lung tissue compared with vehicle-treated mice. Notably, prostacyclin (PGI_2_) BAL concentration was only lowered in celecoxib-treated mice. Series 2: BI 1029539 significantly reduced sepsis-induced BAL inflammatory cell recruitment, lung injury score and lung expression of mPGES-1 and inducible nitric oxide synthase. Treatment with BI 1029539 also significantly prolonged survival of mice with severe sepsis. Anti-inflammatory and anti-migratory effect of BI 1029539 was confirmed in peripheral blood leukocytes from healthy volunteers.

**Conclusions:**

BI 1029539 ameliorates leukocyte infiltration and lung injury resulting from both endotoxin-induced and sepsis-induced lung injury.

**Supplementary Information:**

The online version contains supplementary material available at 10.1186/s12967-021-03016-9.

## Introduction

Acute lung injury (ALI), a common complication of sepsis, involves excessive inflammation and disruption of the alveolar-capillary barrier resulting in lung edema and impaired gas exchange [1–3]. ALI remains a significant source of morbidity and mortality in critically ill patients, underscoring the need for novel therapeutic interventions [4].

Prostaglandin E_2_ (PGE_2_) is involved in various biological processes, including pain, fever, inflammation, angiogenesis, and tumorigenesis, often exerting opposing effects due to its affinity to four PGE_2_ receptor subtypes, PGE_2_ receptors 1–4 [5–8]. PGE_2_ is produced from arachidonic acid via the cyclooxygenase (COX) pathway, with the terminal step catalyzed by PGE synthases (PGES). There are three major PGES isoforms: cytosolic PGES (cPGES), microsomal PGES 1 (mPGES)-1, and mPGES-2 [9]. mPGES-1 is weakly expressed under normal physiological conditions, but strongly up-regulated by proinflammatory stimuli [4, 5] such as interleukin-1β (IL-1β), lipopolysaccharide (LPS) and tissue injury [9–12]. cPGES and mPGES-2 are constitutively expressed [9]. Studies in mPGES-1 knockout mice identified mPGES-1 as being responsible for the excessive PGE_2_ production and thus a key amplifier of acute inflammatory processes [13]. Consequently, mPGES-1 derived PGE_2_ plays an important role in various inflammatory responses commonly displayed in sepsis including swelling, fever, inflammatory pain, and apnea [9–12, 14–17].

Nonsteroidal anti-inflammatory drugs (NSAIDs) alleviate pain and inflammation by inhibition of COX-2. However, NSAIDs exhibit cardiovascular risks due to the inhibition of prostanoids critical for normal physiologic functions, such as COX-2-derived prostacyclin (PGI_2_) [18, 19]. In contrast, mPGES-1 inhibition selectively prevents the mPGES-1-derived synthesis of PGE_2_ only [19, 20] making mPGES-1 a potential novel therapeutic target with reduced cardiovascular risk.

One challenge in inhibitor design and selectivity is that amino acid sequence disparities between human, mouse and rat mPGES-1, and it may have impaired research [21]. BI 1029539 (alternative name: OX-MPI, or GS-248 being used in clinical trials) is a potent and selective, small molecular, non-peptide and orally active inhibitor of human mPGES-1 [22–24]. This compound has no affinity for mice or rat mPGES-1. Using knock-in mice that express human mPGES-1, this study examined the effect of BI 1029539 on endotoxin-induced direct lung injury and sepsis-induced indirect lung injury. The anti-inflammatory responses underlying the protection afforded by mPGES-1 inhibition were also examined in human whole blood and primary cells.

## Materials and methods

### Animals

Animal studies were approved by the Institutional Animal Care and Use Committee at Chonbuk National University and complied with the Korean Animal Welfare Act. Knock-in mice expressing the mPGES1 (Ptges) humanized allele were generated by Boehringer Ingelheim using a similar strategy as previously reported [22, 25] and outlined in Additional file 1. Although the hmPGES-1 protein was weakly expressed in the hmPGES-1 knock-in mice at baseline, it can be strong increased upon exposure to LPS and glutamate, or in diseases condition [22, 25]. In total, 150 homozygous humanized mPGES-1 C57Bl/6 mice (8–12 weeks of age) were used. Age-matched mice were equally distributed among all study groups. Mice were group-housed under controlled conditions (21 ± 1 °C, 12-h light/dark cycle) with free access to water and chow.

### Animal models

**Fig. 1:**
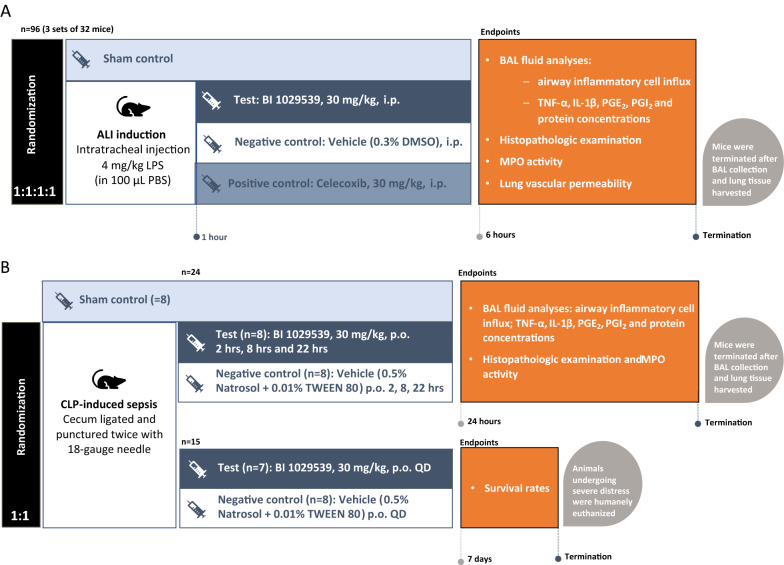
Study schema for **A** LPS-induced acute lung injury and **B** CLP-induced sepsis. ALI, acute lung injury; BAL, bronchoalveolar lavage; DMSO, dimethyl sulfoxide; IL-1β, interleukin 1β; i.p., intraperitoneal; LPS, lipopolysaccharide; MPO, myeloperoxidase; PBS, phosphate buffered saline; PGE_2_, Prostaglandin E_2_; PGI_2_, prostacyclin; p.o., by mouth; QD, once daily; TNF-α, tumor necrosis factor α

Study design for endotoxin-induced lung injury and cecal ligation and puncture (CLP)-induced polymicrobial sepsis models are shown in Fig. 1. Dosing, schedules and output measures are described separately below.

### Endotoxin-induced acute lung injury

Mice were anesthetized with ketamine (80 mg/kg, intramuscular [i.m.]) and xylazine (10 mg/kg, i.m). ALI was induced by intratracheal injection of 4 mg/kg LPS in 100 µl of phosphate-buffered saline (PBS). Mice were randomly assigned to receive intraperitoneal (i.p.) treatment of vehicle (0.3% dimethyl sulfoxide [DMSO]), BI 1029539 (30 mg/kg), or the COX-2 selective NSAID celecoxib (30 mg/kg) 1 h post LPS administration. Mice were anesthetized 6 h post LPS administration and bronchoalveolar lavage (BAL) fluid and lung tissues were collected (as described below).

Three sets of mice were used to obtain the following output measures:

- SET 1: Collection of BAL fluid to measure airway inflammatory cell influx, and tumor necrosis factor (TNF)-α, IL-1β, PGE_2_, and PGI_2_ BAL concentrations, and protein content. Lung tissues were harvested to determine the edema index (wet/dry ratio).
- SET 2: Lung tissues were harvested for histopathological and immunofluorescence examination and myeloperoxidase (MPO) activity.
- SET 3: Evaluation of lung vascular permeability by Evans blue dye method.
  Each set consisted of 32 mice randomly assigned to one of four study groups. Group 1: Sham control; Group 2: LPS + vehicle (negative control group); Group 3: LPS + BI 1029539 (test group); and Group 4: LPS + celecoxib (positive control group).

### Cecal ligation and puncture-induced polymicrobial sepsis

Sepsis was induced in anesthetized mice by CLP as previously described [26]. Briefly, the cecum was ligated and punctured twice with an 18-gauge needle and returned to the abdominal cavity. Sham control animals underwent the same procedure of CLP with the exception that the cecum was neither ligated nor punctured. Mice were resuscitated (5 mL × 100 g^−1^ body weight normal saline subcutaneously) immediately after surgery and returned to their cages. Mice were assigned to one of the following experimental groups:

- CLP sepsis SET 1: Following CLP, mice were randomly assigned to orally receive vehicle (0.5% Natrosol + 0.01% TWEEN 80, n = 8) or BI 1029539 (30 mg/kg, n = 8) at 2, 8, and 22 h after CLP. Sham control received vehicle (0.5% Natrosol + 0.01% TWEEN 80) as well. At 24 h post CLP the mice were re-anesthetized, and BAL fluid and lung tissue samples were collected.
- CLP sepsis SET 2: Survival study, Following CLP, mice orally received twice daily treatments of vehicle (n = 8) or BI 1029539 (30 mg/kg, n = 7). Survival rate was determined through 7 days.

### Bronchioalveolar lavage collection

BAL was collected from anesthetized mice through a 20-gauge angiocath as previously described [27]. Briefly, 0.5 ml of sterile PBS was instilled into the mouse lung and lavaged three times. BAL cell counts were determined using a standard hemocytometer. Differential cell counts were subsequently performed on Giemsa-wright stained (Microscopy Hemacolor-Merck; Germany) cytospin preparations. Cell numbers were standardized/ml of BAL collected and results expressed as number/ml × total volume.

### Histological examination

Formalin-fixed paraffin-embedded tissue was sectioned (5 μm thick), hematoxylin- and eosin-stained, and analyzed by light microscopy. Two sections from one lung were assessed for each mouse, with 10 areas per section analyzed. The degree of lung injury was scored by a trained pathologist, blinded to experimental groups/treatments, using a 5-point scoring system measuring (a) neutrophil infiltration, (b) edema, (c) disorganization of lung parenchyma and (d) hemorrhage, respectively [27]. Higher scores indicate more severe lung abnormalities: 0 = normal, 1 = light, 2 = moderate, 3 = severe, and 4 = very severe [27]. Scores for each of the four categories were combined to provide a total lung injury score (max score 16).

### Immunofluorescence

Details of the primary and secondary antibodies used are provided in Additional file 1: Table S1. Briefly, lung Sections (5 μm) underwent optimal heat-induced epitope retrieval (incubation in 10 mM sodium citrate buffer at 99 °C for 20 min) and were incubated with primary antibody overnight. Primary antibodies were detected following a 1-h incubation in either a FITC or Alexa fluor 594 conjugated secondary antibody. Nuclei were counterstained with 4′,6-diamidino-2-phenylindole contained within the aqueous Ultra Cruz Mounting Medium (sc-24941, Santa Cruz Biotechnology). Omission of primary or secondary antibodies from staining protocol were used as negative controls (data not shown).

### Fluorescent microscopy

Digital micrographs were obtained using a Nikon Eclipse TE2000-U fluorescence microscope (Nikon Corp., Tokyo, Japan) equipped with a Nikon LWD 0.52 digital camera. Fluorescent intensity was quantified using Image Pro Premier 9.1 software, camera settings were maintained for capturing all images. Ten fields of view per sample were quantified. The mean fluorescence measured with in counted number of positive cells/field.

### Biochemical measurements

Total BAL fluid protein concentration was determined using a Smart BCA Assay Kit (Intron Biotechnology Inc. South Korea). Enzyme immunoassay kits for mouse IL-1β (BioLegend, San Diego, CA), TNF-α (R & D Systems, Minneapolis, MN), PGE_2_ and metabolite of PGI_2_, 6-keto prostaglandin F_1α_ (both Cayman Chemical, MI) were used to determine BAL fluid concentrations of these meditators. Neutrophil accumulation in the lung was determined by MPO activity as previously described [28, 29]. MPO activity in each sample was determined by measuring the change in absorbance at 460 nm. Each sample was tested in triplicate. One unit of MPO activity is the amount of enzyme that will reduce 1 µM peroxide per min.

### Lung permeability

Vascular permeability was assessed using Evans blue dye [27]. Briefly, Evans blue dye (20 mg/kg) was administered into the tail vein 30 min before termination. Mice were anesthetized, and the lungs perfused free of blood with PBS containing 5 mM EDTA via thoracotomy with cardiac reperfusion. One part of the lung tissue was dried, and this was standardized across animals. Evans blue was extracted from *en bloc* lung harvests with formamide [27] and the optical density at 620 nm determined. Evans blue dye concentration was calculated from a standard curve.

### Human peripheral blood

#### Cell migration assay

Neutrophils and monocytes were isolated from 10 healthy donor peripheral blood samples by ficoll density gradient centrifugation [30] and stimulated with TNFα (5 ng/ml) in the presence or absence of BI 1029539 (0.01, 0.1, 1, 10 µM) for 24 h. Treated cells (1 × 10^6^ cells in 0.5 ml serum free-RPMI medium) were added to the upper chamber of the transmigration plate (3 μm pore size for neutrophils, 8 μm pore size for monocytes; Thermo Scientific, Waltham, MA) and 1.5 ml of serum free-RMPI media containing the same concentrations of TNFα and the BI 1029539 as in the upper chambers were added to the lower chambers. Cells were incubated at 37 °C and 5% CO_2_ for 24 h. Transmigrated cells were collected from the lower chamber and quantified by hemocytometer. Four replicates per test condition were performed and replicate averages presented.

#### Ex vivo whole blood assay for cytokines

Whole blood from 10 healthy donors was diluted 1:1 with serum free RPMI 1640 medium and treated with LPS (0.1 ng/ml) in the presence or absence of BI 1029539 (0.001, 0.01, 0.1, 1 µM) and incubated for 24 h at 37 °C and 5% CO_2_. After 24 h, samples were centrifuged for 10 min at 12,000 g and 4 °C. Cell-free supernatants were collected and stored at − 80 °C. Enzyme immunoassay kits were used to determine the supernatant concentrations of TNF-α (detection range 15.6–1,000 pg/ml, PeproTech, Rocky Hill, NJ) and IL-1β (detection range 3.90–250 pg/ml, R & D Systems, Minneapolis, MN). Three replicates per test condition were performed and replicate averages presented.

### Statistical analysis

All data are reported as mean ± SEM. Between-group differences were determined by analysis of variance for repeated measures followed by Bonferroni’s post hoc test using GraphPad Prism 5. P values < 0.05 were considered significant. Survival estimates were determined by Kaplan–Meier analysis.

## Results

### BI 1029539 preserves lung architecture and reduces immune cell influx into the lungs of LPS-challenged mice

**Fig. 2:**
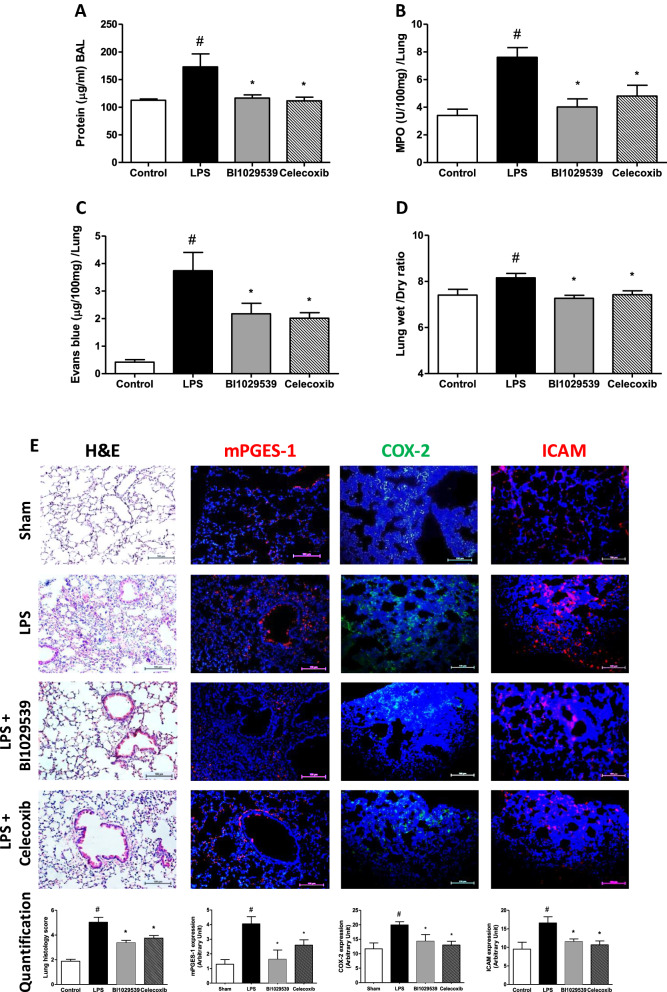
BI 1029539 reduces LPS-induced lung inflammation and tissue damage. **A**–**D** Treatment with BI 1029539 reduced BAL protein content, Lung MPO activity, vascular leakage and water content, compared to vehicle controls at 6 h after LPS administration in mPGES-1 knock-in mice. **E** BI 1029539 treatment reduced LPS-induced histological lung damage (H&E) and lung expression of mPGES-1 (red color), COX-2 (green color) and ICAM-1 (red color) compared with vehicle controls at 6 h after administration of LPS in mice. Blue color: DAPI staining of cell nuclei in tissue. All values are mean ± SEM, n = 6–8. #p < 0.05 vs sham, * < 0.05 vs vehicle-treated LPS group. COX-2, cyclooxygenase-2; H&E, hematoxylin and eosin; ICAM, intracellular adhesion molecule; LPS, lipopolysaccharide; mPGES-1, microsomal prostaglandin-E synthase; MPO, myeloperoxidase

Intratracheal LPS injection resulted in a marked increase in lung permeability as evidenced by a significant increase in BAL fluid protein content as well as by vascular Evans blue leakage into the lungs (Fig. 2A, C). BAL fluid protein content and vascular leakage induced by LPS were significantly attenuated after treatment with BI1029539 and celecoxib, respectively (Fig. 2A, 3C). There was a decrease in myeloperoxidase (MPO) activity and edema index (wet/dry ratio) in lung tissues of BI1029539-treated as well as celecoxib-treated mice (Fig. 2B, D). Intratracheal LPS injection-induced ALI characterized by destruction of lung architecture, a marked increase in lung permeability, and excessive inflammatory cell infiltration, compared with the sham control group (Fig. 2E). Destruction of lung architecture was characterized by interstitial edema and neutrophil accumulation and resulted in significantly increased lung histology scores (Fig. 2E). All LPS-induced pathological changes were attenuated in mice treated with BI 1029539 and celecoxib. Additionally, immunofluorescence demonstrated increased mPGES-1, COX-2 and intracellular adhesion molecule-1 (ICAM-1) expression within the lung parenchyma following intratracheal LPS injection in lungs of vehicle-treated mice (Fig. 2E). These increases in mPGES-1, COX-2 and ICAM-1 were attenuated by both BI 1029539 and celecoxib (Fig. 2E).

**Fig. 3:**
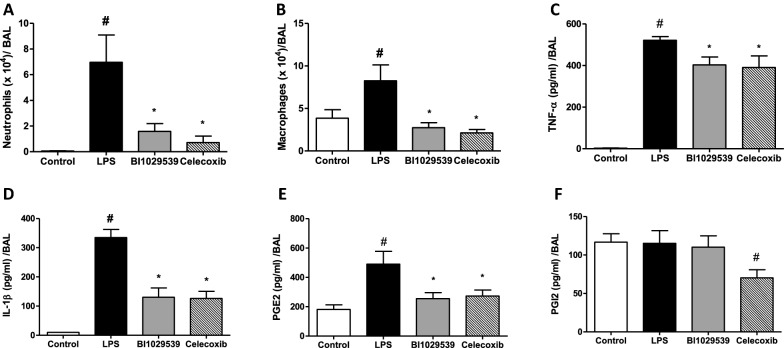
BI 1029539 reduces LPS-induced airway inflammation. Treatment with BI 1029539 reduced LPS-induced lung inflammatory cell accumulation and PGE_2_ production in bronchoalveolar lavage (BAL) fluid at 6 h after LPS administration in mPGES-1 knock-in mice. **A** Neutrophils, **B** Macrophages, **C** TNF-α, **D** IL-1β, **E** PGE_2_, and **F** PGI_2_. The metabolite of PGI_2_, 6-keto prostaglandin F_1α_, was used as a surrogate for PGI_2_ concentration. All values are mean ± SEM, n = 6–8. #p < 0.05 vs sham, *p < 0.05 vs vehicle-treated LPS group. BAL, bronchoalveolar lavage; IL-1β, interleukin 1β; PGE_2_, Prostaglandin E_2_; PGI_2_, prostacyclin; LPS, lipopolysaccharide; TNFα, tumor necrosis factor α

Inflammatory cell influx into the airways as demonstrated by increased cell numbers in BAL fluid post LPS injection was accompanied by elevated levels of TNF-α, IL-1β and PGE_2_ (Fig. 3). Cell numbers were standardized/ml BAL recovered. BAL recovery volume range was 1.4–1.5 ml. The number of total cellular infiltrates, neutrophils and macrophages in the BAL fluid was reduced by 80.7%, 93.6% and 67% respectively, in mice treated with BI 1029539, compared with 78.6%, 93.5% and 74% reduction in mice treated with celecoxib (Fig. 3A, B). Furthermore, BAL TNF-α, IL-1β and PGE_2_ concentrations were significantly lower in mice treated with BI 1029539 and celecoxib, compared with vehicle controls (Fig. 3C–E). PGI_2_ concentration, as determined by 6-keto prostaglandin F_1α_, was significantly reduced by celecoxib only (Fig. 3F).

### BI 1029539 attenuates CLP-induced lung injury and prolongs survival

**Fig. 4:**
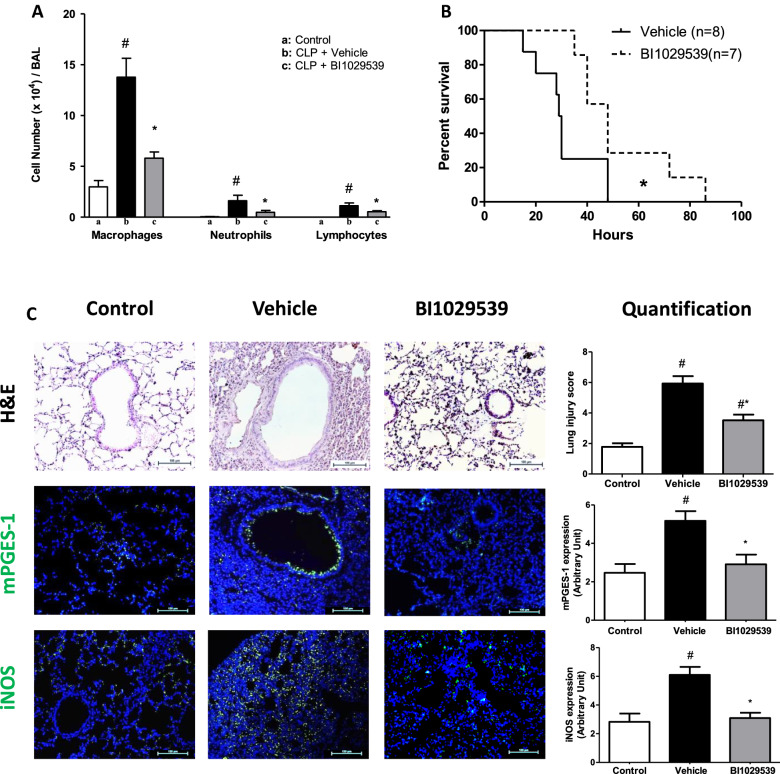
BI 1029539 reduces sepsis-induced lung inflammation and tissue damage. Treatment with BI 1029539 reduced **A** inflammatory cell count at 24 h after CLP, and **B** prolonged survival following CLP in mPGES-1 knock-in mice. Treatment with BI 1029539 reduced CLP-induced **C** histological lung damage, and the lung expression of mPGES-1 (green color) and iNOS (green color). Blue color: DAPI staining of cell nuclei in tissue. All values are the mean ± SEM. N = 6–8. #p < 0.05 vs the sham control group, *p < 0.05 vs the vehicle treated-CLP group. BAL, bronchoalveolar lavage; CLP, cecal ligation and puncture; H&E, hematoxylin and eosin; iNOS, inducible nitric oxide synthase; mPGES-1, microsomal prostaglandin-E synthase

CLP-induced marked lung damage and a significant increase in total BAL cell numbers (Fig. 4A) predominantly driven by an influx of macrophages and lymphocytes (Fig. 4A). Treatment with BI 1029539 significantly reduced sepsis-induced lung macrophage recruitment (Fig. 4A). Importantly, BI 1029539 improved CLP-induced mortality, prolonging mice survival vs vehicle treatment (Fig. 4B).

Histological evaluation revealed a marked reduction of CLP-induced tissue alteration following BI 1029539 treatment (Fig. 4C) translating into lower lung injury scores vs vehicle treatment (Fig. 4C). Expression of inflammatory tissue markers, mPGES-1 and inducible nitric oxide synthase (iNOS), were increased by CLP and their expression attenuated by BI 1029539 (Fig. 4C).

### BI 1029539 reduces human peripheral blood monocyte and neutrophil migration and inhibits LPS-induced cytokine production

**Fig. 5:**
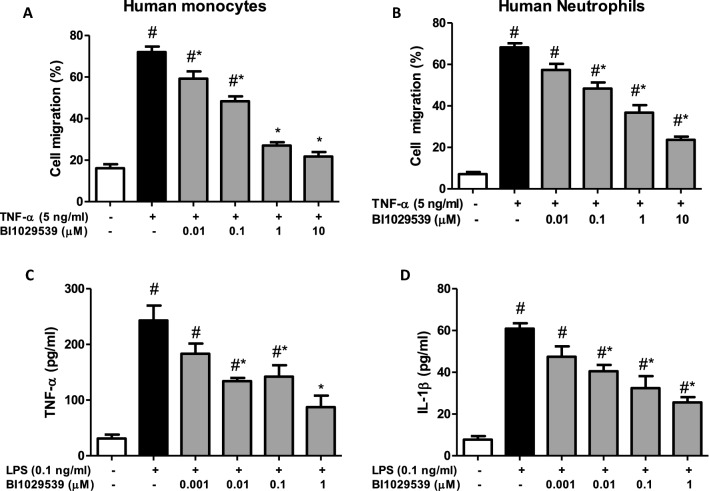
BI 1029539 reduces human peripheral blood inflammatory cell migration and inflammatory mediator release. **A**, **B** mPGES-1 inhibition with BI 1029539 reduced TNFα-induced human monocyte and neutrophil migration. Data are mean ± SEM, n = 4, #p < 0.05 vs the control, *p < 0.05 vs the vehicle group. (C-D) mPGES-1 inhibition with BI 1029539 reduced LPS-induced TNF-α and IL-1β production in human peripheral blood. Data are mean ± SEM, n = 5–6, #p < 0.05 vs Control, *p < 0.05 vs the vehicle group. IL-1β, interleukin 1β; LPS, lipopolysaccharide; mPGES-1, microsomal prostaglandin-E synthase; TNFα, tumor necrosis factor

To assess whether mPGES-1 inhibition directly affects immune cell migration, the impact of BI 1029539 on human blood monocytes and neutrophils transmigration in vitro was assessed. BI 1029539 attenuated TNF-α-induced monocyte and neutrophil migration in a dose dependent fashion (Fig. 5A, B). Consistent with the idea of a direct effect on immune cells, BI 1029539 reduced LPS-induced TNFα and IL-1β production in human peripheral blood (Fig. 5C, D).

## Discussion

Acute inflammation and disruption of vascular integrity are key features of ALI, contributing to the high morbidity and mortality associated with this condition. Using two in vivo models we show that BI 1029539 significantly attenuates pulmonary inflammation, alveolar-capillary leakage, edema formation, and lung injury resulting directly from endotoxin-induced ALI and indirectly via CLP-induced sepsis. BI 1029539 significantly reduced lung neutrophil infiltration, BAL levels of TNF-α and IL-1β, and BAL protein concentration after intratracheal injection of LPS. Moreover, BI 1029539 also prolonged mice survival following CLP-induced severe polymicrobial sepsis.

Uncontrolled recruitment of neutrophils into the lung interstitium and alveolar space is a pathologic hallmark of ALI [1, 2, 27] and correlates with disease severity and poor outcome. Reduced epithelial cell barrier function facilitates neutrophil migration and the influx of macromolecules and protein-rich fluid into the alveoli causing impaired cell fluid transport, edema formation, and lung injury [31–33]. Inhibition of mPGES-1 has been shown to attenuates efficient resolution of acute inflammation by enhancing CX3CL1 expression in mice [34], and suppressed the synthesis of PGE2, but not other prostaglandins inhibitable by nonsteroidal anti-inflammatory drugs (NSAIDs), yet retained NSAID-like efficacy at inhibiting lipopolysaccharide-induced pyresis, hyperalgesia, and iodoacetate-induced.

Osteoarthritic pain in mice [25]. Here, BI 1029539 reduced neutrophil accumulation within the lungs of LPS-challenged mice, consistent with data from mPGES-1 knockout models demonstrating a key role for mPGES-1 in mediating neutrophil recruitment to sites of inflammation [35–38]. Furthermore, we confirmed that indirect inflammatory lung injury induced by polymicrobial sepsis was associated with excessive lung macrophage recruitment [27], as well as a marked up-regulation of tissue mPGES-1. BI 1029539 markedly reduced sepsis-induced lung macrophage recruitment and lung injury, and prolonged survival of mice following severe sepsis. Collectively the data suggests BI 1029539 inhibition of mPGES-1 represents a potential therapeutic target for neutrophilic and mPGES-1-driven inflammatory conditions, like ALI and sepsis that warrants further investigation.

In patients with ALI, the extent of BAL neutrophil accumulation correlates with disease severity and poor outcome. Conversely, neutrophil depletion reduces lung injury [39]. Deletion of mPGES-1 in experiment models has demonstrated an important role in polymorphonuclear neutrophil (PMN) recruitment to sites of inflammation [35–38]. Across animal models of differing inflammatory conditions, deletion of mPGES-1 reduced neutrophil infiltration, attenuated cytokine production and tissue destruction, and decreased pain sensitivity [36–38, 40]. In the present study, intratracheal administration of LPS elicited lung injury that was associated with neutrophil infiltration and a marked up-regulation of mPGES-1. BI 1029539 significantly reduced LPS-induced neutrophil influx, lung edema and vascular leakage, and protected alveolar-capillary barrier integrity.

Consistent with our previous report, we found that indirect lung inflammatory injury induced by polymicrobial sepsis was associated with excessive lung macrophage recruitment [27], as well as a marked up-regulation of tissue mPGES-1. mPGES-1 inhibition with BI 1029539 significantly reduced polymicrobial sepsis-induced lung macrophage recruitment and lung injury. Importantly, treatment with BI 1029539 significantly prolonged survival of mice following severe sepsis. It is noteworthy to mention that BI1029539 and Celecoxib treatment did not completed block the cell recruitment and TNF-α release, or lung injury. This finding may suggest the severity of this disease and the involvement of various components in disease process. Factors other than mPGES-1 and iNOS may be involved in this multi-factorial disease process of lung injury.

The protective effect afforded by BI 1029539 in reducing lung injury was accompanied by reduced expression of the inducible proinflammatory enzymes COX-2, mPGES-1 and iNOS, as well as the generation of proinflammatory cytokines and ICAM expression. Inflammatory stimuli induce PGE_2_ production through inducible COX-2 and mPGES-1 [18]. Selective COX-2 inhibitors are associated with an increased cardiovascular risk, which is largely attributed to suppression of the cardioprotective properties of COX-2-derived PGI_2_ and PGD_2_ biosynthesis [35, 41]. Inhibition of mPGES‐1 reduced noradrenaline‐induced vasoconstriction in human blood vessels by increasing PGI_2_ synthesis [42, 43]. In a preclinical study, GS-248 completely inhibited LPS induced PGE2 formation in whole blood [23]. In urine, GS-248 reduced PGE2 and increased PGI2, while celecoxib reduced both PGE2 and PGI2 metabolites [23]. These findings suggest that selective inhibition of mPGES-1 results in systemic shunting of PGH2 to PGI2 formation, leading to anti-inflammatory and vasodilatory effects, while preventing platelet activation [23]. In the present study, PGE_2_ production was equally suppressed by COX-2 as well as mPGES-1 inhibition with celecoxib and BI 1029539, respectively, providing similar anti-inflammatory efficacy profiles. Importantly, PGI_2_ production was only reduced in mice treated with celecoxib. Findings from the present study further support the concept that selective mPGES-1 inhibitors may have the potential to become a distinct class of novel anti-inflammatory agents that act by selectively suppressing inflammatory PGE_2_ formation, but not other prostaglandins suppressed by COX-2 inhibitors linked to increased cardiovascular risk.

Pro-inflammatory cytokines such as TNFα and IL-1β, are involved in the early phases of ALI, elevated both systemically (plasma) and locally (BAL), and are predictive of clinical outcome [44]. It is postulated that endotoxin simulation of resident alveolar macrophages generates much of the IL-1β and TNF-α initiating an inflammatory cascade whereby neighboring cells produce a battery of chemokines and ICAMs that mediate the alveolar recruitment of neutrophils, monocytes and lymphocytes [1, 2, 44]. In the present study, BI 1029539 reduction of LPS-induced neutrophil influx and lung injury was accompanied by the local reduction of TNF-α and IL-1β, and ICAM-1 expression. Furthermore, BI 1029539 reduced LPS-stimulated cytokine production in human blood and inhibited human neutrophils and monocytes migration in vitro. These findings demonstrate that mPGES-1 inhibition with BI 1029539 can protect from LPS- or CLP-induced lung injury by inhibition of leukocytes recruitment and down-regulation of inflammatory mediators.

## Conclusion

mPGES-1 is the critical enzyme downstream of COX-2 through which PGE_2_ is formed while it is not involved in the generation of PGI_2_ [19, 32]. We demonstrate that mPGES-1 inhibition with BI 1029539 ameliorates endotoxin and sepsis-induced lung injury, and importantly, improves survival following severe polymicrobial sepsis. This protective effect was observed without affecting PGI_2_ levels. As such, mPGES-1 inhibitors exhibit a COX-2 inhibitor-like efficacy profile but may be devoid of COX-2 inhibitor-associated negative cardiovascular outcomes. Our data suggests mPGES-1 represents a valuable therapeutic target for ALI and sepsis-related lung injury with potential therapeutic advantage over selective COX-2 inhibitors which warrants further investigation.

## Supplementary Information

**Additional file 1**: **Table S1. **Immunofluorescence antibody details.

## Data Availability

The datasets used and/or analyzed during the current study are available from the corresponding author on reasonable request.
