# Supplementary material for: Inhibition of microsomal prostaglandin E synthase-1 ameliorates acute lung injury in mice
Source: J Transl Med. 2021 Aug 9;19:340. doi: 10.1186/s12967-021-03016-9 (PMC8351447; doi:10.1186/s12967-021-03016-9)
Supplement: Supplementary file 1 — Additional file 1: Table S1. Immunofluorescence antibody details. [file 12967_2021_3016_MOESM1_ESM.docx]

**Inhibition of Microsomal Prostaglandin E Synthase-1 Ameliorates Acute Lung Injury in Mice**

Malarvizhi Gurusamy, PhD^1^, Saeed Nasseri, PhD^1^, Dileep Reddy, PhD^1^, Huiying Feng, MA^1^, Dongwon Lee, PhD^1^, Anton Pekcec, PhD^2^, Henri Doods, PhD^2^, and Dongmei Wu, PhD^1, 3^

^1^Department of BIN Convergence Technology, Chonbuk National University, Jeonju, South Korea; ^2^Boehringer Ingelheim Pharma GmbH & Co. KG, Biberach an der Riss, Germany; ^3^Department of Research, Mount Sinai Medical Center, Miami Beach, FL, USA

# Supplementary Material

**METHODS**

**Generation of transgenic humanized mPGES-1 C57Bl/6 mice**

Transgenic mice constitutively expressing the mPGES-1 (Ptges) humanized allele were generated by Boehringer Ingelheim using a similar strategy as previously reported (21). Briefly, the mouse mPGES-1 (Ptges) genomic region encompassing exon 1 (starting at the translation initiation site), intron 1 and exon 2 were replaced with an engineered human mini-gene (full-length human PTGES cDNA was engineered by inserting human PTGES intron 1 between exons 1 and 2). An additional polyadenylation signal was inserted downstream of exon 3 which contains the 3´ untranslated region (3´ UTR) to improve the expression of the human PTGES cDNA in mouse cells and prevent downstream transcription of the remaining mouse gene. Mouse genomic sequences downstream of exon 2 were left intact, to keep all potential regulatory elements driving expression of the mPGES-1 (Ptges) gene. A positive selection marker (PuroR) was flanked by F3 recombination sites and inserted downstream of the human PTGES mini-gene. A targeting vector was generated using bacterial artificial chromosome (BAC) clones from the C57BL/6J RPCI-23 BAC library and was transfected into the TaconicArtemis C57BL/6N Tac ES cell line. Homologous recombinant clones were isolated using positive (puromycin resistance) and negative (thymidine kinase) selections and the humanized allele was finalized after Flp-mediated removal of the selection marker. Human PTGES protein was expressed under the control of the endogenous mouse mPGES-1 (Ptges) promoter. Due to removal of mouse mPGES-1 (Ptges) exons 1 and 2, and to termination of transcription at the inserted 3´UTR and polyadenylation signals within the human mini-gene, mouse mPGES-1 (Ptges) protein was no longer expressed. The C57BL/6N ES cell line was grown on a mitotically inactivated feeder layer comprised of mouse embryonic fibroblasts in Dulbecco’s Modified Eagle’s Medium (DMEM) high glucose medium containing 20% FBS (PAN-Biotech GmbH, Aidenbach, Germany) and 1200 U/mL leukemia inhibitory factor (ESGRO^®^ Recombinant Mouse LIF Protein, ESG1107, MilliporeSigma, Burlington, MA). 107 cells and 30 μg of linearized DNA vector were electroporated (Gene Pulser Xcell Electroporation System, Bio-Rad Laboratories, Hercules, CA) at 240 V and 500 μF. Puromycin selection (1 μg/mL) started on Day 2, counterselection with ganciclovir (2 μM) started on Day 5 after electroporation. ES clones were isolated on Day 8 and analyzed by Southern blotting after expansion and freezing of clones in liquid nitrogen. After administration of hormones, superovulated BALB/c female mice were mated with BALB/c male mice. Blastocysts were isolated from the uterus at 3.5 days postcoitus (dpc). For microinjection, blastocysts were placed in a drop of DMEM with 15% FCS under mineral oil. A flat tip, piezo-actuated microinjection-pipette with an internal diameter of 12-15 μm was used to inject 10-15 targeted C57BL/6NTac ES cells into each blastocyst. After recovery, eight injected blastocysts were transferred to each uterine horn of 2.5 dpc, pseudopregnant NMRI female mice. Chimerism was measured in chimeras (G0) by coat color contribution of ES cells to the BALB/c host (black/white). Highly chimeric mice were bred to strain C57BL/6 females. Germline transmission was identified by the presence of black, strain C57BL/6 offspring (G1). Mice were backcrossed to the C57BL/6 background strain over at least six generations.

**Supplementary Table 1.** Immunofluorescence antibody details

| **Antibody** | **Supplier** | **Catalogue number** |
| --- | --- | --- |
| *Primary antibodies* |  |  |
| Rabbit anti-mouse COX-2 | Santa Cruz Biotechnology, Santa Cruz, CA | sc-7951 |
| Rabbit anti-mouse iNOS | Santa Cruz Biotechnology | sc-651 |
| Rabbit anti-mouse ICAM-1 | Santa Cruz Biotechnology | sc-1511 |
| Rabbit anti-human mPGRS-1 | Cayman Chemical, Ann Arbor, Michigan, USA | 160140 |
| *Secondary antibodies* |  |  |
| FITC conjugated goat anti-rabbit IgG | Santa Cruz Biotechnology | sc-2012 |
| Alexa Fluor 594 conjugated goat anti-rabbit IgG (preabsorbed) | Abcam, Cambridge, MA, USA | ab 150084 |

COX-2, cyclooxygenase 2; FITC, fluorescein isothiocyanate; ICAM-1, intracellular adhesion molecule-1; IgG, immunoglobulin G; iNOS, inducible nitric oxide synthase; mPGRS-1, microsomal prostaglandin E synthase.
